# Double-Bundle Anterior Cruciate Ligament Reconstruction With Primary Repair and Hamstring Autograft Augmentation

**DOI:** 10.1016/j.eats.2022.12.021

**Published:** 2023-04-16

**Authors:** Samuel K. Van de Velde

**Affiliations:** Department of Orthopedic Surgery, Columbia University Irving Medical Center, New York, New York, U.S.A.

## Abstract

The optimal treatment of a ruptured anterior cruciate ligament (ACL) restores the patient’s native anatomy and biomechanics as close to normal as possible. The purpose of this technical note is to describe an ACL reconstruction technique in which a double-bundle concept is used, wherein one bundle contains repaired ACL tissue and the second bundle contains a hamstring autograft, and each bundle is tensioned independently. Even in chronic cases, this technique allows for incorporation of the native ACL because, more often than not, there is sufficient tissue of good quality available for repair of one bundle. By augmenting the ACL repair with an autograft sized to the fit the individual anatomy, the patient’s ACL tibial footprint can be closely restored to normal, and the potential benefits of tissue preservation are combined with the biomechanical strengths of an autograft double-bundle ACL reconstruction.

## Introduction

Single-bundle autograft reconstruction is the standard of care for a complete rupture of the anterior cruciate ligament (ACL) in young, active patients. However, single-bundle ACL reconstruction fails to normalize knee biomechanics and does not prevent osteoarthritis.^1^ Even short-to-midterm patient-reported and radiographic outcomes have been similar between some young, active patients who were treated with rehabilitation alone and those who underwent single-bundle ACL reconstruction.^2^ The quest for the optimal ACL reconstruction is, therefore, ongoing. Anatomic double-bundle ACL reconstruction was introduced to approximate the patient’s original anatomy more closely and, thereby, improve the postoperative kinematics. Despite the biomechanical advantage in early laboratory studies,^3^ double-bundle ACL reconstruction has not replaced single-bundle reconstruction as the gold standard. The double-bundle technique is technically challenging, requires more autograft tissue, and its early clinical outcomes have not been overwhelmingly superior to single-bundle reconstruction. Primary ACL repair is a promising alternative to single-bundle ACL reconstruction. ACL repair restores the ligament’s original anatomy and (conceivably) its biomechanical and proprioceptive function. Unfortunately, ACL repair is limited to a small subset of patients—those with acute, proximal avulsions of the ACL. Even in these select cases, the failure rate following ACL repair with suture augmentation was 10 times higher compared to ACL reconstruction in adolescents.^4^ Biological augmentation of the repair with a protein implant decreased the failure rate, but the repair remained restricted to acute injuries with at least 50% of the length of the ACL attached to the tibia.^5^ In acute cases with insufficient remnant tissue, hamstring augmentation has been performed to create a more robust single-bundle ACL repair,^6^^,^^7^ reminiscent of the primary ACL repair techniques with semitendinosus augmentation performed in the early 1990s.^8^ The purpose of this technical note was to describe an ACL reconstruction technique in which a double-bundle concept is used wherein the first bundle contains repaired ACL tissue, and the second bundle contains a hamstring autograft, and each bundle is tensioned independently.

## Surgical Technique

In this technique description (Video 1), the posterolateral (PL) bundle is repaired and the anteromedial (AM) bundle of the ACL is reconstructed. The surgical steps for AM reconstruction and PL repair are not shown in the present technique description, but follow the same principles as outlined below. The decision on which bundle to repair and which bundle to reconstruct depends on the individual ACL remnant characteristics (Table 1).

**Table 1:** Measurement of ACL Characteristics

Preoperative MRI	Knee Arthroscopy
• The length of the ACL tibial footprint:Measure from the most anterior to the most posterior fiber of the ACL tibial attachment site on the sagittal image with the largest exposure of ACL fibers at the tibial attachment site	• The size of the original ACL tibial footprint • The size and location of the ACL remnant footprint • The length of the ACL remnant
• The width of the ACL tibial footprint:Measure between the most medial and lateral portions of the ACL tibial attachment site on the coronal image	• The quality of the remnant tissue

ACL, anterior cruciate ligament; MRI, magnetic resonance imaging.

### Surgical Approach

Preoperative MR imaging is used to measure the ACL tibial footprint (Table 1). Following general anesthesia and bilateral knee examination, arthroscopy is performed with standard anterolateral (AL) and AM portals; any meniscal and/or cartilaginous pathology is addressed.

**Fig 1:**
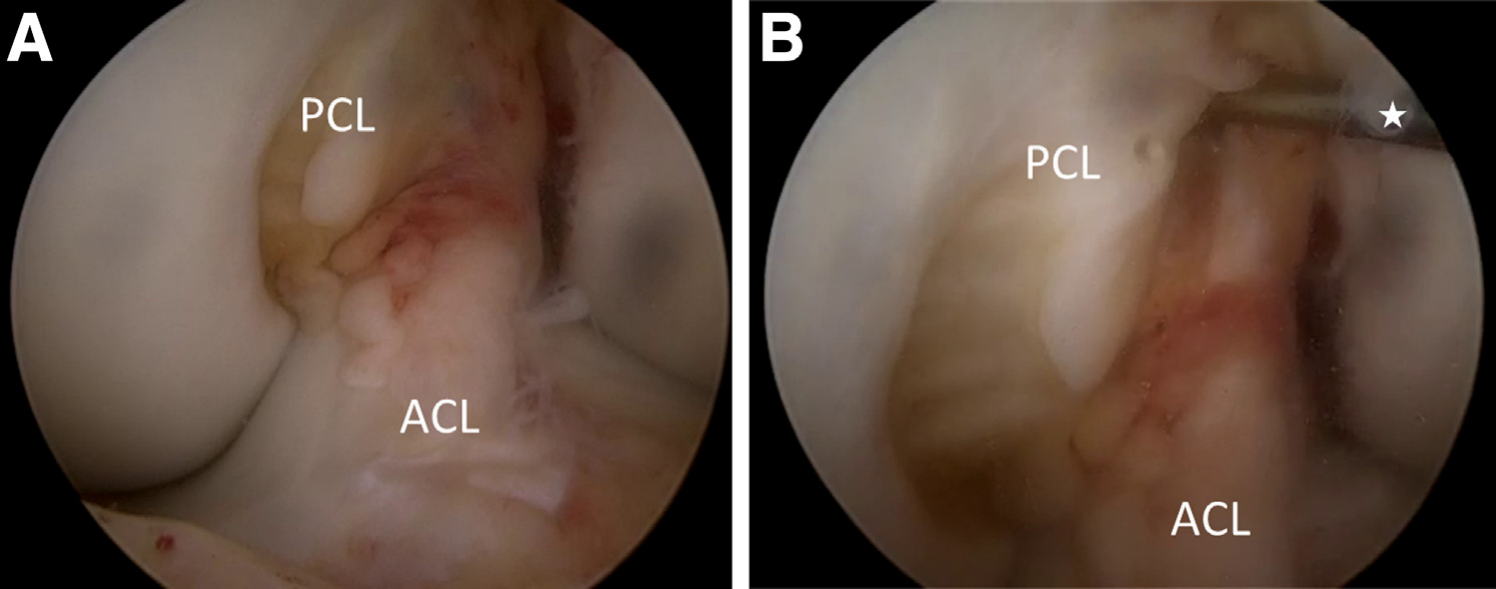
Intraoperative images showing the evaluation of ACL tear characteristics and partial release of ACL remnant. (A) Arthroscopic view through the AM portal showing how very often, the ACL remnant is scarred down onto the PCL. (B) Arthroscopic view through the AM portal showing the partial release of the ACL remnant with probe (star). Arthroscopic scissors and shaver are used for the release as well (not shown; left knee). ACL, anterior cruciate ligament; AM, anteromedial; PCL, posterior cruciate ligament.

**Fig 2:**
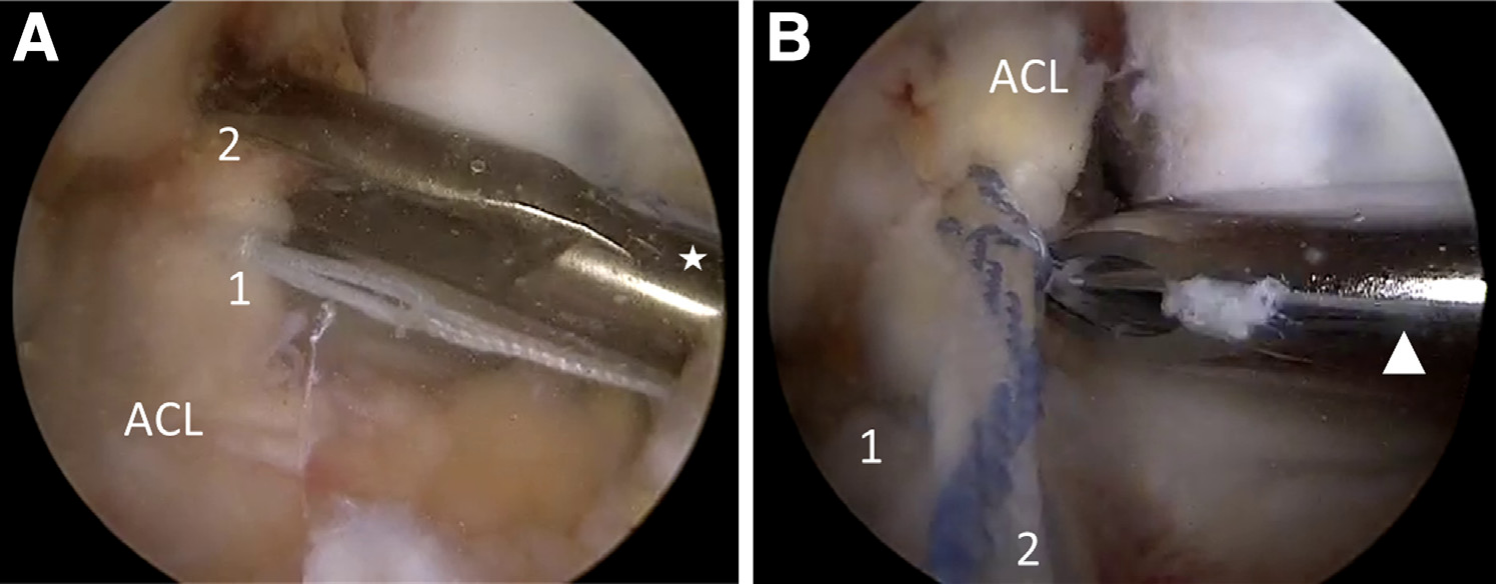
Intraoperative images showing the placement of repair sutures and complete release of ACL remnant (left knee). (A) Arthroscopic view through the AM portal showing the placement of a 25-mm FiberRing (*1*) with preattached shuttle loop (Arthrex, Naples, FL) at the base of the ACL remnant with a shoulder Scorpion suture passer (star) (Arthrex). A 1.3-mm SutureTape (Arthrex) is placed more proximally in the remnant (*2*). (B) Arthroscopic view through the AM portal showing how with tension on the repair sutures (*1* and *2*), the release of the ACL remnant is completed with arthroscopic scissors (not shown) and shaver (triangle). ACL, anterior cruciate ligament; AM, anteromedial.

Switching between AM and AL portals, the ACL tear characteristics are evaluated using a probe (Table 1, Fig 1A). A partial release of the ACL remnant is performed with a combination of probe, arthroscopic scissors, and prudent use of a shaver (Fig 1B). On the basis of the remnant characteristics, either AM or PL bundle is repaired. In the present technique description, the PL bundle is repaired, and the AM bundle is reconstructed. A shoulder Scorpion suture passer (Arthrex, Naples, FL) is used to place a 25-mm FiberRing with preattached shuttle loop (Arthrex) at the base of the ACL remnant (Fig 2A). For additional fixation strength and maximum approximation of the remnant to the femoral wall to avoid cyclops formation, a 1.3-mm SutureTape (Arthrex, Naples, FL) is placed more proximally in the remnant. The FiberRing shuttle loop and SutureTape ends are then docked out of the AM portal. With tension on these sutures, the ACL remnant is now completely released from its surroundings, leaving its tibial attachment intact (Fig 2B).

The semitendinosus tendon is harvested in the standard fashion. On the basis of the preoperative MR imaging and intraoperative measurements, the graft is either doubled, tripled, or quadrupled, and, if necessary, supplemented with a gracilis tendon, so that the combined final graft diameter plus the remnant tibial footprint diameter approximate that of the native ACL tibial footprint. The graft is secured with TightRope RT devices (Arthrex).

**Fig 3:**
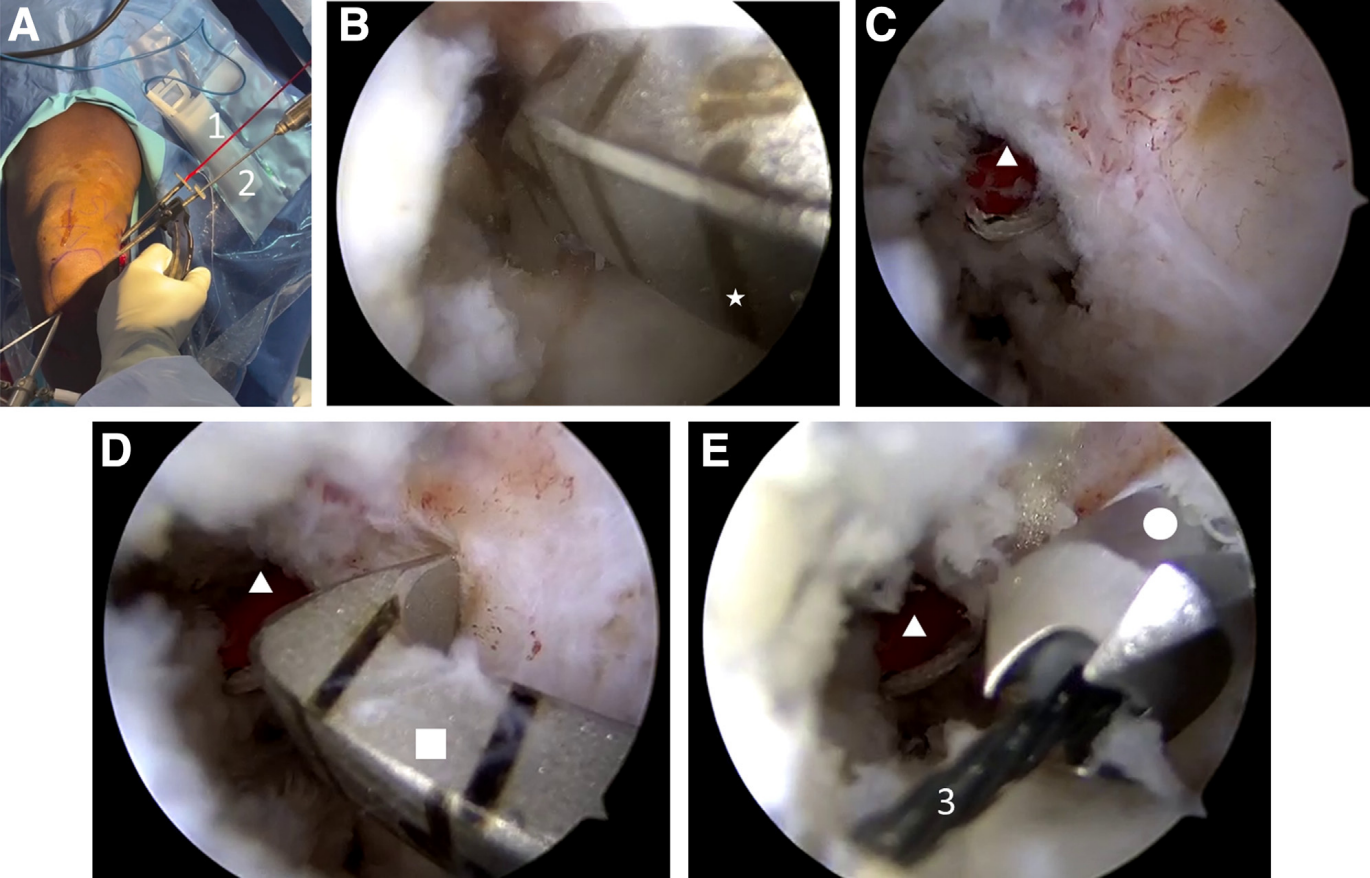
(A) Intraoperative photograph of a left knee secured in a knee holder with the patient supine, showing the creation of a socket at the femoral AM location (*1*) and a tunnel at the femoral PL location (*2*) in an outside-in fashion. (B) Arthroscopic view through the AM portal showing the femoral drill guide (star), entered through the AL portal, positioned at the anatomic location of the femoral AM attachment. A FlipCutter (Arthrex, Naples, FL) is advanced outside-in (not shown), opened to the diameter of the graft, and a 20-mm socket is created in a retrograde fashion. (C) Arthroscopic view through the AM portal showing a cannulated stick (triangle) with draw suture (FiberStick, Arthrex) advanced into the socket. (D) Arthroscopic view through the AM portal showing a point-to-point drill guide (square), which is entered through the AL portal and is used to advance a 3.0-mm cannulated drill outside-in at the location of the PL femoral attachment site. (E) Arthroscopic view through the AM portal showing how, after removal of the central stylus from the cannulated drill (circle), a nitinol loop (*3*) is advanced through the cannula. The cannulated stick (triangle) is visible at the femoral AM location (left knee). AM, anteromedial; AL, anterolateral; PL, posterolateral.

**Fig 4:**
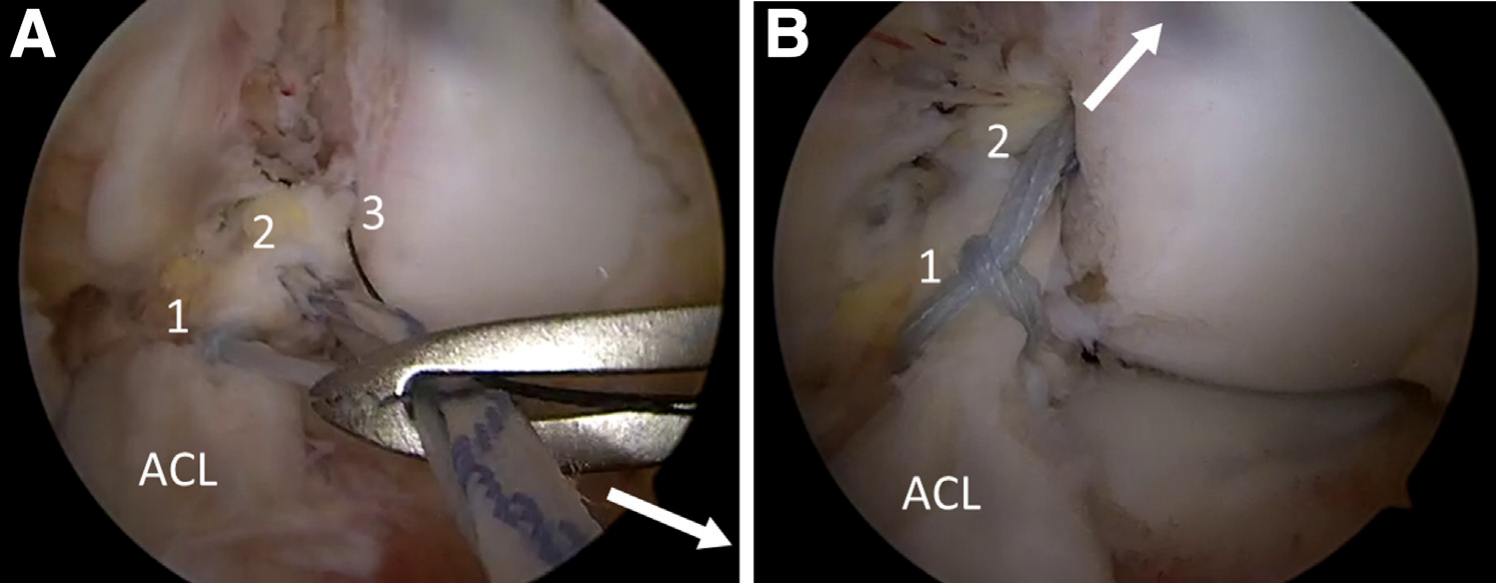
Intraoperative images showing the advancement of ACL repair sutures. (A) Arthroscopic view through the AM portal showing the shuttle loop attached to the FiberRing (*1*), the 1.3-mm SutureTape (*2*), and the nitinol loop (*3*) (Arthrex, Naples, FL) being pulled outside the knee through the AL portal to prevent skin bridges. (B) Arthroscopic view through the AM portal showing the ACL repair sutures (*1* and *2*) shuttled through the femoral tunnel using the nitinol loop (not visible anymore) (left knee). ACL, anterior cruciate ligament; AM, anteromedial; AL, anterolateral.

Looking from the AM portal, the femoral drill guide is entered through the AL portal and positioned at the anatomic location of the femoral AM attachment (Fig 3, A and B). A longitudinal incision is placed over the lateral aspect of the distal femur, with sharp incision through the fascia. A FlipCutter (Arthrex) is advanced outside-in, opened to the diameter of the graft, and a 20-mm socket is created in a retrograde fashion at the location of the AM femoral attachment. A cannulated stick with draw suture (FiberStick; Arthrex) is advanced into the socket (Fig 3C). The FiberStick, draw suture, and drill sleeve are left in place to avoid tunnel convergence. The location of the native femoral PL attachment is defined. A point-to-point drill guide is entered through the AL portal (Fig 3D), and a 3.0-mm cannulated drill is drilled outside-in at the location of the PL femoral attachment site. The central stylus is removed from the cannulated drill and a nitinol loop is advanced through the cannula (Fig 3E). The repair sutures within the ACL remnant, together with the nitinol loop, are first pulled outside the knee through the AL portal and then shuttled through the femoral tunnel using the nitinol loop (Fig 4).

**Fig 5:**
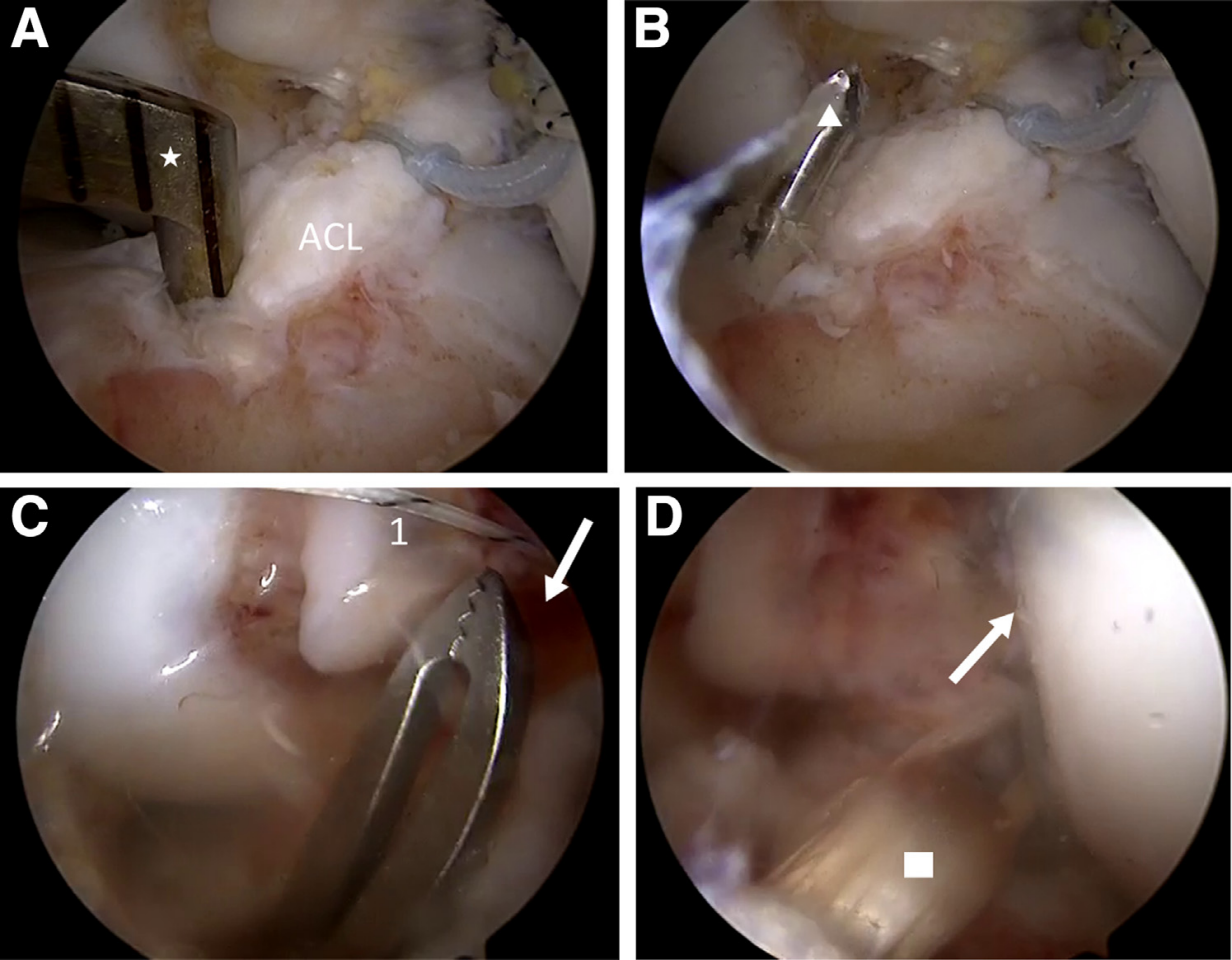
Intraoperative views showing the creation of a tunnel at the tibial AM location and the advancement of a hamstring autograft. (A) Arthroscopic view through the AL portal showing a point-to-point drill guide (star) entered through the AM portal and positioned at the anatomic location of the tibial AM attachment. (B) Arthroscopic view through the AL portal showing the advancement of a guide pin (triangle) through the center of the anatomic AM bundle attachment of the native ACL. The guide pin is then overdrilled with a reamer with the size determined by the graft diameter. (C) Arthroscopic view through the AL portal showing the femoral draw suture in the femoral AM socket (*1*) being pulled outside the knee through the tibial tunnel. (D) Arthroscopic view through the AL portal showing the hamstring graft (square) being docked into the femoral socket using the femoral draw suture (left knee). ACL, anterior cruciate ligament; AL, anterolateral; AM, anteromedial.

Looking from the AL portal, a guide pin is advanced from the anteromedial aspect of the tibia using the hamstring harvesting incision through the center of the anatomic AM bundle attachment of the native ACL (Fig 5, A and B). The guide pin is overdrilled with a reamer with the size determined by the graft diameter. The femoral draw suture that was left in place in the femoral AM socket is now pulled outside the knee through the tibial tunnel (with care to stay anterior to the ACL repair sutures in the PL femoral tunnel) (Fig 5C). Using the femoral draw suture, the surgeon docks the graft into its femoral socket (Fig 5D).

**Fig 6:**
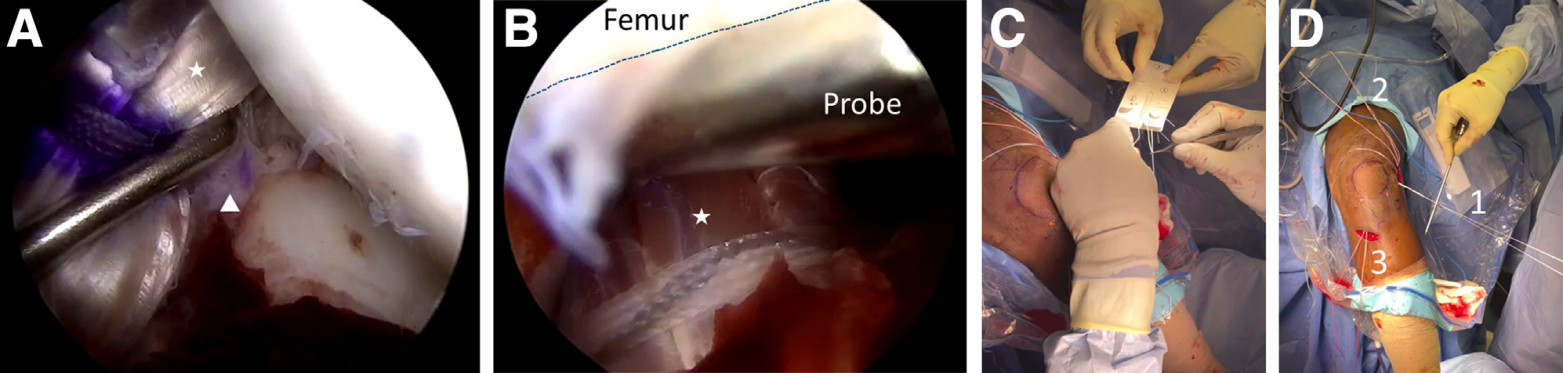
(A) Arthroscopic view through the AL portal showing the individual tensioning of the hamstring graft (star) and ACL repair (triangle). The graft is tensioned using the TightRope RT with extension button for tibial fixation, with the knee in extension plus a combined posterior and external rotation vector. (B) Arthroscopic view through the AM portal showing that notchplasty is not required since full extension of the knee can be achieved with the probe positioned between the femoral notch and graft. (C) Intraoperative photograph of a left knee secured in a knee holder with the patient supine shows the attachment of the FiberRing to an ACL Repair TightRope implant (Arthrex, Naples, FL) using the sequential passing sutures on the assembly card and the SutureTape ends in the proximal ACL stump passed through the empty lateral eyelets of the button. (D) Intraoperative photograph of a left knee secured in a knee holder with the patient supine and with the knee in extension and neutral rotation, showing the tensioning of the ACL repair using the TightRope cinch stitches (*1*) followed by tying of the SutureTape ends over the button (not shown). (*2*), Femoral draw sutures of hamstring graft; (*3*), tibial draw sutures of hamstring graft (left knee). ACL, anterior cruciate ligament.

After cycling of the knee, the graft is tensioned using the TightRope RT with extension button for tibial fixation, with the knee in extension plus a combined posterior and external rotation vector (Fig 6, A and B). The FiberRing within the ACL remnant is attached to an ACL Repair TightRope implant (Arthrex, Naples, FL) using the sequential passing sutures on the assembly card (Fig 6C). The SutureTape ends in the proximal ACL stump are passed through the empty lateral eyelets of the button. With the knee in extension and neutral rotation, the ACL repair is tensioned using the TightRope cinch stitches, and the SutureTape ends are tied over the button (Fig 6D).

### Postoperative Management

A standardized ACL reconstruction protocol under the guidance of a physical therapist is prescribed. Weight bearing is as allowed as tolerated unless meniscal repair was performed. A brace is not routinely used.

## Discussion

The concept of ACL repair with autograft augmentation is not new. Open repair of an acute ACL injury with semitendinosus tendon augmentation, often supplemented with a lateral extra-articular reinforcement, provided good to excellent results in active patients.^8^ However, with the advent of arthroscopically assisted ACL reconstruction, only few surgeons continued augmented ACL repair (and lateral extra-articular tenodesis). Despite improved clinical outcomes and decreased morbidity compared to the open ACL surgical techniques, graft failure and persistent rotational instability were also seen in patients after single-bundle autograft ACL reconstruction.

Preservation of ACL remnant during ACL reconstruction was found to enhance graft revascularization and integration, with greater initial graft coverage with ACL remnant tissue, resulting in greater postoperative stability. Primary ACL repair results in the greatest degree of remnant preservation, and with advances in fixation and tensioning devices, its use has seen a resurgence. However, even when the repair was restricted to adolescents with acute, proximal avulsions of the ACL, failure rate of ACL repair with suture augmentation was significantly increased relative to ACL reconstruction.^4^ To provide additional strength to the ACL repair— especially when insufficient tissue for a complete repair is present—primary repair has been augmented with a single-bundle autograft, resulting in a single-bundle ACL construct containing both preserved and reconstructed tissue.^6^^,^^7^ In these augmented repairs, the central portion of the ACL remnant has to be debrided to allow for graft placement, and the lack of rotational control seen in single-bundle ACL reconstruction might persist.

The current surgical technique incorporates the novel ACL repair methods in a double-bundle procedure, wherein one bundle contains repaired ACL tissue, and the second bundle contains a hamstring autograft, and each bundle is tensioned independently. The advantage of this technique is that the possible benefits of ACL remnant preservation are combined with the biomechanical advantages of two individually tensioned bundles for the control of rotational laxity. The technique is less challenging than a four-tunnel, two-graft double-bundle reconstruction. However, the technique is undeniably more demanding than a standard single-bundle ACL reconstruction, with decreased visibility due to obstruction of the preserved ACL tissue, risk of femoral tunnel convergence, and intricate suture management. Nevertheless, these technical challenges can be minimized as detailed in Table 2. Clinical follow-up studies of the technique are needed to determine whether the added complexity results in improved outcomes compared to the standard single-bundle ACL reconstruction.

**Table 2:** Pearls and Pitfalls

• Frequently, especially in more chronic cases, the bulk of the ACL remnant is stuck down onto the PCL.
• Placement of the repair sutures in the ACL remnant is easier if a partial release is performed initially with some proximal remnant tissue left attached. Once the repair sutures are secured, tension on these sutures helps with the complete release of the ACL remnant.
• A shoulder Scorpion suture passer (Arthrex, Naples, FL) works better than the knee suture passer for penetrating the ACL remnant tissue at its base to place the 25-mm FiberRing (Arthrex, Naples, FL).
• For additional fixation strength and maximum approximation of the remnant to the femoral wall to avoid cyclops formation, a 1.3-mm SutureTape (Arthrex) is placed more proximally in the ACL remnant.
• The ACL remnant can make visibility of the femoral wall cumbersome. When entering the femoral drill guide, an assistant maintains tension of the sutures placed in the ACL remnant and docked out of the AM portal to improve visibility.
• To avoid tunnel convergence during drilling of the femoral PL repair tunnel, the FiberStick, draw suture, and drill sleeve of the AM femoral socket are left in place.
• To avoid damage to the ACL remnant during tibial tunnel drilling, the reamer is advanced in an oscillating manner through the proximal tibial cortex, while an assistant holds some tension on the repair sutures.
• Suture management is critical during this procedure. Stay anterior to the ACL repair sutures when pulling the ACL graft femoral draw suture outside the knee through the tibial tunnel.
• Irrigation with saline over the ACL Repair TightRope implant (Arthrex) assembly card helps with pulling the leader suture through the button.

ACL, anterior cruciate ligament; AM, anteromedial; PCL, posterior cruciate ligament; PL, posterolateral.

The current surgical concept can be individualized to the patient’s ACL anatomy and surgeon’s preference. The described steps could be followed to incorporate a quadriceps or bone-patellar tendon-bone autograft instead of a hamstring autograft, or semitendinosus tendon with its tibial insertion left attached, into the double-bundle ACL.

In conclusion, by augmenting the ACL repair with an autograft sized to the fit the individual anatomy, the patient’s ACL tibial footprint can be closely restored to normal and the potential benefits of tissue preservation are combined with the biomechanical strengths of an autograft double-bundle ACL reconstruction.

## Supplementary Data

ICMJE author disclosure forms

Video 1 Step-by-step technique description of the double-bundle anterior cruciate ligament reconstruction with primary repair and hamstring autograft augmentation (left knee). Informed consent was obtained from the subject and parent.
